# Advances in the Applications and Studies of Polyurethane Foam for Flexible Strain Sensors

**DOI:** 10.3390/polym17131851

**Published:** 2025-07-02

**Authors:** Shuai Huang, Guanbing Liu, Ying Sun, Xiacong Zhang

**Affiliations:** 1 School of Materials Science and Engineering, East China University of Science and Technology, Shanghai 200237, China; 2 Department of Polymer Materials, School of Materials Science and Engineering, Shanghai University, Shanghai 200444, China

**Keywords:** polyurethane foam, strain sensor, flexible sensors

## Abstract

Polyurethane (PU) foam, renowned for its structural versatility, elasticity, compressibility, and adaptability, has garnered significant attention for its use in flexible strain sensors due to its capability to detect mechanical deformation. This review presents a comprehensive analysis of both the studies and recent advancements in PU foam-based strain sensors, particularly those incorporating conductive materials. The review begins by examining the chemical composition and structural characteristics of PU foam, followed by a discussion of various fabrication methods and their effects on sensor performance. It also explores the sensing mechanisms, including piezoresistive, piezoelectric, and capacitive effects. Moreover, key applications in motion detection, health monitoring, and environmental and industrial sensing are examined. Finally, the review addresses technological advancements, current challenges, and prospects.

## 1. Introduction

Over the past decade, the demand for stretchable and wearable strain sensors has surged, driven by their applications in health monitoring, human motion detection, gesture recognition, smart electronics, and environmental and structural sensing [1,2]. These flexible sensors are typically fabricated by integrating conductive materials, such as carbonaceous, metallic fillers, or conductive polymers, into stretchable polymeric substrates. This combination enhances electrical conductivity and mechanical properties, yielding sensors with superior performance and adaptability.

Unlike traditional metal or semiconductor-based strain sensors, flexible sensors offer greater stretchability, broader strain response ranges, and higher sensitivity [3,4]. They maintain stable electromechanical properties under significant strain, converting mechanical stimuli like tension or pressure into electrical signals through physical structural changes in the sensing elements.

High-performance strain sensors require excellent sensitivity, a wide strain response range, stability, and precise detection capabilities. Recent studies have shown that flexible sensors can meet these demands, particularly when incorporating porous structures that provide low density and exceptional compressibility across large strain regions [5]. These three-dimensional (3D) conductive networks can be readily tuned by external mechanical deformations, enabling diverse sensing behaviors [6].

In addition to polyurethane (PU) foam, other materials, such as polydimethylsiloxane (PDMS) [7] and rubber [8], have also been explored for flexible strain sensor applications. PDMS, for example, is widely used due to its high stretchability and biocompatibility, while silicone rubber offers excellent durability and elasticity. Conductive polymers such as polypyrrole (PPy) and polyaniline (PANI), and carbon-based materials like graphene and carbon nanotubes (CNTs), are commonly integrated into these substrates to improve the sensors’ electrical performance [9]. These materials have different advantages depending on the specific application, ranging from high stretchability for wearable electronics to enhanced sensitivity and response range for health monitoring and structural sensing. However, challenges such as optimizing the balance between stretchability and conductivity, as well as ensuring long-term stability, remain.

Among the materials explored for these sensors, polyurethane (PU) foam stands out due to its unique properties [10,11]. Its porous 3D cellular structure creates a lightweight, compressible matrix that detects minute mechanical deformations. With a low elastic modulus and the ability to withstand significant deformation, PU foam enhances pressure sensitivity. Compared to thin films and colloids, it exhibits superior compressibility, flexibility, and elastic response, making it well-suited for applications requiring conformity to the human body or complex geometries [12]. Its exceptional elasticity ensures durability through repeated deformation cycles without significant performance loss, while its biocompatibility and versatility in scalable, cost-effective fabrication techniques enable integration into wearable devices for motion and healthcare monitoring [13], human–machine interfaces [14,15], and soft robotics [16].

**Figure 1:**
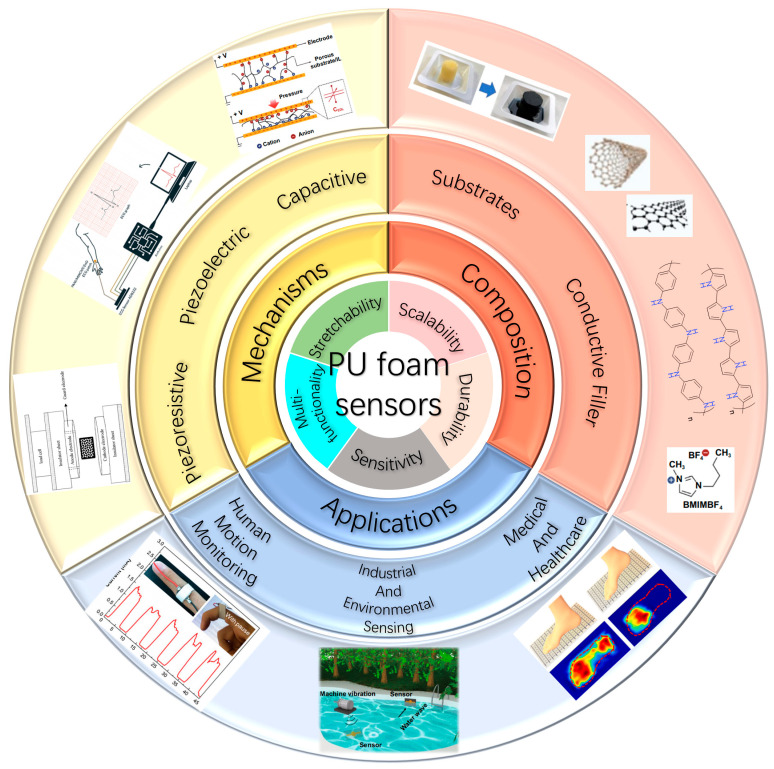
Polyurethane foam-based flexible strain sensors and potential applications in motion detection, health monitoring, industrial, and environmental sensing. Reprinted with permission from refs. [17,18,19,20,21,22,23,24].

This review examines the development of PU foam-based flexible strain sensors, focusing on the incorporation of conductive materials (Figure 1). The integration of conductive fillers into the inherently insulating PU foam matrix is essential for transforming it into a functional strain sensor capable of transducing mechanical deformation into measurable electrical signals. The selection of appropriate conductive materials is therefore paramount, directly influencing critical sensor performance metrics. We analyze the fundamental composition and properties of PU foam-based sensors, key sensing mechanisms, and fabrication techniques. Applications, including motion detection, health monitoring, and industrial and environmental sensing, are explored. The review concludes by addressing technological advances, current challenges, and promising research directions relevant to both researchers and industry professionals.

## 2. Fundamental Composition of PU Foam-Based Sensor

### 2.1. PU Foam Substrates

Polyurethane (PU) foam is a versatile material that can be produced in a variety of forms and applications. Its composition is mainly derived from the reaction between polyols (compounds containing hydroxyl groups) and isocyanates (compounds containing isocyanate groups), forming urethane linkages. This basic structure provides the foundation for the material’s wide range of properties, which can be tailored to suit specific applications such as flexible, rigid, thermoplastic, or even waterborne PUs [25].

The polyol component plays a significant role in determining the foam’s characteristics. Polyols can be petroleum-based, such as glycerine, which contains primary hydroxyl groups, or bio-based, such as castor oil, which contains secondary hydroxyl groups. The reaction between these polyols and isocyanates results in different physical and mechanical properties. For instance, a mixture of primary and secondary hydroxyl groups can be used to reduce the reliance on petroleum-derived polyols, which also influence the reactivity and physical properties of the foam. Additionally, the incorporation of various additives and catalysts, such as surfactants and blowing agents, further modifies the foam’s structure. Surfactants, for example, help in controlling cell size, stabilizing the foam structure, and improving the overall mechanical properties. These surfactants can be nonionic or cationic and are often used to prevent collapse during the foaming process, ensuring that the final foam is durable and stable. By adjusting these parameters—such as the types and amounts of polyols, isocyanates, and catalysts—PU foam can be precisely engineered for a wide range of applications from thermal insulation to biomedical devices [11].

PU foams are crucial for developing flexible strain sensors due to their customizable mechanical properties, open-cell structures, and compatibility with conductive fillers. These foams can be obtained through commercial sources or laboratory synthesis, with each method offering unique advantages for sensor fabrication [23].

#### 2.1.1. Commercially Available PU Foams

Commercially available PU foams are widely used in flexible strain sensors due to their easy accessibility and seamless integration [26]. The key parameter for selection is the pores per inch (PPI), which affects the foam’s mechanical properties and suitability for specific applications. Higher PPI values indicate a denser foam with smaller pores, offering increased rigidity, while lower PPI values result in softer and compressible foams for applications requiring flexibility. Lower PPI foams (e.g., 10–20 PPI) are preferable, whereas higher PPI foams (e.g., 60–100 PPI) provide better structural support for sensors needing mechanical strength and sensitivity [27]. Additionally, the base material—whether polyether or polyester—should be selected based on the environmental conditions the sensor will face, such as humidity and temperature variations.

#### 2.1.2. Laboratory Preparation of PU Foams

Laboratory preparation offers the ability to tailor PU foam properties precisely, allowing customization of mechanical, thermal, and conductive characteristics for specific sensor needs. The preparation methods fall into two main categories:

1. Direct Foaming from Isocyanates and Polyols

**Figure 2:**
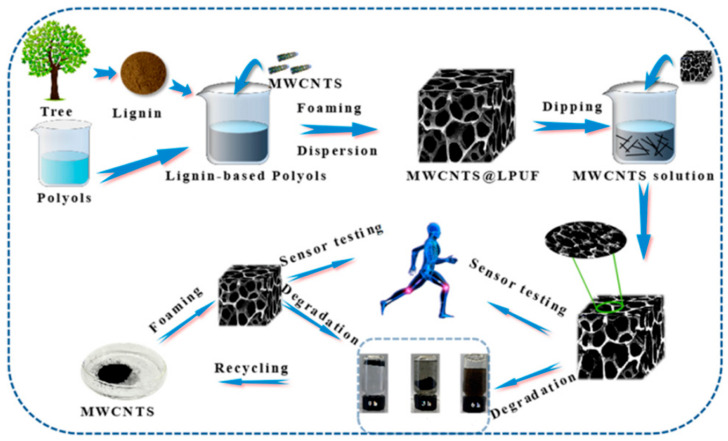
Schematic illustration of the fabrication process of lignin-based PU foam for a strain sensor. Reprinted with permission from ref. [28].

This method synthesizes PU foams through step-growth polymerization between isocyanate and polyol monomers. Different foaming methods, such as chemical (water), physical (hydrocarbons, halocarbons), and supercritical CO_2_ foaming, enable customization based on the specific monomer structures and ratios. By adjusting the types and ratios of surfactants, catalysts, and blowing agents, properties can be optimized, and conductive fillers can be incorporated during foaming for uniform distribution. Water-blown foaming, an eco-friendly technique, uses water to generate CO_2_, facilitating conductive filler integration while maintaining good mechanical properties. Additionally, lignin-based polyols derived from plant biomass offer a sustainable alternative, providing enhanced mechanical and thermal properties suited for environmentally friendly strain sensors. (Figure 2) [24].

2. Thermoplastic Polyurethane (TPU) Foams

**Figure 3:**
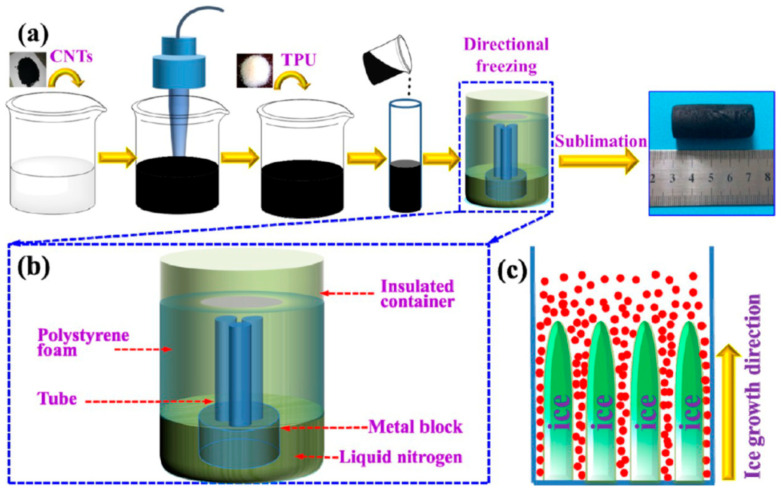
(**a**) Aligned CNT/TPU foam production, (**b**) Directional-freezing apparatus schematic diagram, and (**c**) the directional-freezing process. Reprinted with permission from ref. [29].

Thermoplastic Polyurethane (TPU) is a versatile polymer known for its elasticity, abrasion resistance, and chemical stability. Composed of alternating hard and soft segments, TPU’s properties can be tailored by adjusting the ratios of diisocyanates, polyols, and chain extenders, making it ideal for flexible and durable strain sensors [9]. TPU foams can be prepared in the lab using methods that allow precise control over the foam’s microstructure, which is crucial for optimizing sensor performance. Two prominent techniques are template-assisted synthesis and phase separation methods:

- Template-Assisted Method:

This method involves adding a sacrificial template with a TPU solution. After solidification, the template is removed, leaving behind a porous TPU structure. The pore size and distribution can be controlled by selecting appropriate templates, enabling the fabrication of foams with specific characteristics tailored to sensor applications. For instance, the directional freezing method creates strain sensors with aligned pore structures, balancing mechanical strength and conductive network stability [29] (Figure 3).

b. Phase Separation Techniques:

Thermally Induced Phase Separation (TIPS) [30,31]: In TIPS, a polymer solution is cooled, inducing phase separation that leads to the formation of a porous structure. The cooling rate and polymer concentration can be adjusted to control the pore morphology, which is essential for achieving the desired mechanical and electrical properties in strain sensors.

Water-Vapor-Induced Phase Separation (WVIPS) [32]: WVIPS is similar to TIPS but utilizes exposure to water vapor to induce phase separation. This method can be advantageous for processing temperature-sensitive materials and allows for the creation of foams with distinct pore structures that can enhance the performance of flexible strain sensors.

The choice between commercial and laboratory-synthesized PU foams depends on specific strain sensor requirements. Commercial foams provide convenience and consistency for large-scale production, while laboratory synthesis offers precise control over properties like pore structure, mechanical strength, and conductivity. This flexibility allows researchers and manufacturers to develop sensors that meet exact specifications for various applications.

### 2.2. Conductive Filler

#### 2.2.1. Carbon Nanotubes

**Figure 4:**
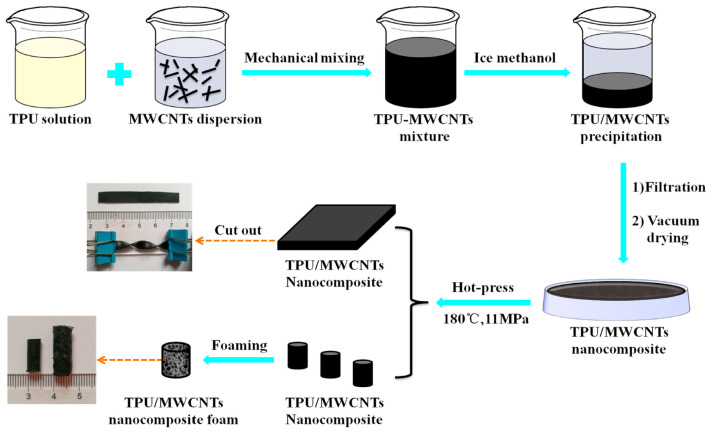
TPU/MWCNTs composite and composite foam preparation. Reprinted with permission from ref. [33].

Carbon nanotubes (CNTs) are cylindrical nanostructures formed by carbon atoms organized into a hexagonal lattice. They exhibit exceptional mechanical strength, electrical conductivity, and thermal stability, making them ideal for reinforcing materials in flexible electronics and strain sensors. As conductive fillers in PU foam-based strain sensors, CNTs enhance both electrical conductivity and mechanical properties (Figure 4). Their high aspect ratio and conductivity form conductive networks within the PU matrix, enabling the sensor to detect strain through resistance changes [17,33].

When incorporated into PU foam, CNTs create conductive porous foams, suitable for applications which need great sensitivity and mechanical flexibility. These composites excel in pressure, temperature, and gas detection because of their high surface area, conductivity, and flexibility [5]. However, incorporating CNTs into PU foam presents challenges, primarily due to their tendency to clump together through van der Waals forces, which lead to poor dispersion throughout the polymer matrix. This uneven distribution can compromise the uniformity and performance of the composite [34]. CNTs are dispersed in solvents (e.g., ethanol, DMF) using ultrasonication, and then deposited on PU foam via immersion and drying cycles. Additionally, CNTs’ inert surfaces interact weakly with the polymer matrix, leading to suboptimal mechanical and electrical properties. Achieving proper CNT dispersion and alignment requires specialized techniques, which increases manufacturing complexity and cost.

Several methods can improve CNT dispersion in PU foam. Surface modification through chemical treatments enhances CNT-matrix compatibility, while ultrasonic treatment helps break up CNT clusters. Pre-dissolving CNTs in appropriate solvents can also aid integration. Firstly, the dry MWCNTs were dispersed in the *n*-hexane solvent to ensure full wetting. Subsequently, room temperature vulcanized silicone rubber (RTVSR) and compatibilizer were added to the solution, and mechanical stirring was carried out for 1 h to form a uniform doped solution. Finally, immerse the PU foam into the solution to allow the solution to fully penetrate the foam pores. During this process, MWCNTs are coated and fixed on the surface of the PU foam pore walls by RTVSR to form a conductive network. Meanwhile, the compatibilizer enhances the interfacial bonding between MWCNTs and the rubber phase [23]. Foaming PU with CNTs present promotes better distribution and stronger bonding. Carbon nanotubes (CNTs) are integrated into polyurethane (PU) foam through a two-step salt-templating and electrostatic adsorption method. The foam is immersed in an aqueous dispersion of carboxyl-functionalized multi-walled CNTs, where electrostatic interactions between cationic quaternary ammonium groups on the PU backbone and anionic carboxyl groups on the CNTs drive robust adsorption. The foam is then dried at 60 °C to yield the conductive composite (PU/MWCNTs foam). This approach ensures uniform CNT distribution, enhanced interfacial adhesion (validated by post-sonication conductivity stability), and high strain sensitivity (GF = 5.2 at <30% strain) for wearable sensors [28]. These dispersion improvements make CNT-enhanced PU foam more effective for flexible strain sensors across various applications.

#### 2.2.2. Graphene

**Figure 5:**
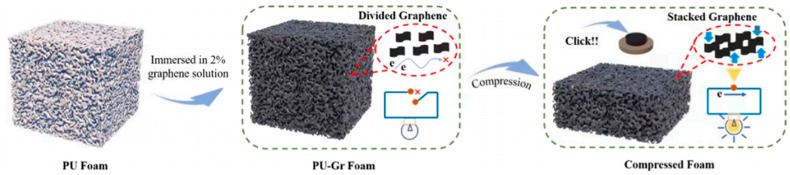
Schematic diagram of the preparation process and sensing mechanism of rGO/PU foam. Reprinted with permission from ref. [36].

Graphene is a single layer of carbon atoms arranged in a two-dimensional honeycomb lattice. It possesses outstanding mechanical strength, electrical and thermal conductivity, and remarkable flexibility, making it highly attractive for use in electronics, energy storage, and sensors (Figure 5) [35,36]. Graphene’s exceptional properties significantly enhance the overall performance of polymer composites by improving mechanical strength, electrical conductivity, and thermal properties [37].

Graphene as a conductive filler faces two main challenges. First, its high surface area causes agglomeration at higher concentrations, reducing mechanical properties [38]. Second, the stacking of graphene sheets limits its conductivity [36]. While manufacturers use stabilizers to improve dispersion, this could negatively affect the conductivity of foam sensors. The stacking of graphene sheets limits its conductivity [39].

While these processes effectively reduce graphene oxide, they may reduce the intrinsic properties of materials, complicating manufacturing and reducing effectiveness. Researchers have explored simpler alternatives, such as avoiding chemical treatments or modifying the composite’s structure. A promising approach involves combining multi-walled carbon nanotubes with rGO flakes to enhance the sensitivity and conductivity of the sensor [22].

#### 2.2.3. MXenes

**Figure 6:**
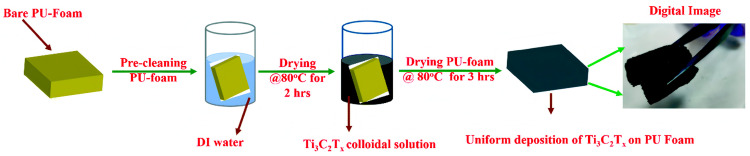
The typical Ti_3_C_2_T_x_ thin film deposition on the flexible PU-foam substrate schematic procedure by using the dip-coating technique. Reprinted with permission from ref. [6].

MXenes, which are developing rapidly, are two-dimensional materials with great potential for flexible strain sensors. Their chemical structure follows the formula M_n+1_X_n_T_x_, where M is an early transition metal, X stands for carbon or nitrogen, and T_x_ represents surface functional groups (O, OH, and F), with n ranging from 1 to 3. MXenes are created by selectively etching A-group elements from MAX phase precursors, resulting in materials with great electroconductivity, hydrophilicity, and a high aspect ratio, thus enabling them excellent for sensor applications [6] (Figure 6).

Among MXenes, Ti_3_C_2_T_X_ is the most extensively studied. It is produced by etching aluminum from Ti_3_AlC_2_, with functional groups (OH, O, and F) replacing the aluminum, which imparts hydrophilicity to the material. This property enhances their dispersibility in water and other polar solvents, facilitating the creation of uniform composites (Figure 6) [40]. The functional groups also improve interaction with polymers and conductive fillers, enhancing sensor performance.

MXenes offer several advantages in flexible strain sensors. Their high electrical conductivity makes them excellent conductive fillers, improving sensor sensitivity and response time. Their hydrophilic nature enhances integration with polymer matrices, improving mechanical properties and dispersion. Additionally, their high aspect ratio and surface area strengthen interactions with the polymer matrix, further boosting sensor performance. However, the cost of synthesizing high-quality MXenes can limit their commercial adoption.

#### 2.2.4. Liquid Metals (LMs)

Liquid metals (LMs) are low-melting-point alloys that have emerged as promising materials for flexible strain sensors due to their unique properties. These materials combine the high electrical conductivity of metals with the flexibility and stretchability of liquids, which are able to flow and adapt to mechanical changes—stretching, bending, or compression [41]. Unlike traditional rigid conductive fillers (such as solid metals or carbonaceous materials) that often compromise stretchability and elasticity, liquid metals’ fluidity allows them to redistribute and fill voids under stress [42]. This ensures the conductive path remains intact and functional.

Their low viscosity also enables seamless integration into the polymer matrix, ensuring uniform dispersion and consistent conductivity throughout the sensor. However, some liquid metals, especially gallium-based alloys, pose safety risks, including toxicity and skin irritation [43]. These metals require special handling precautions. Despite their chemical inertness, exposure to air or extreme temperatures may affect their long-term stability, and environmental factors like oxidation can impact performance over time.

#### 2.2.5. Intrinsically Conductive Polymers (ICPs)

**Figure 7:**
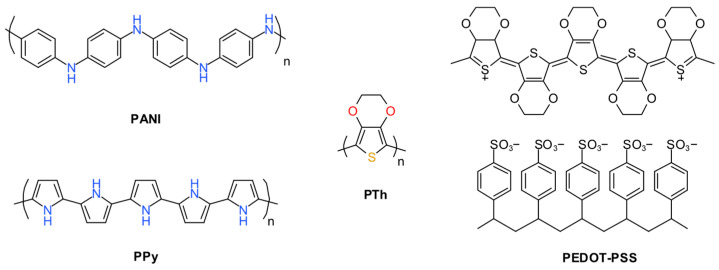
Structures of PANI, PPy, PTh, and PEDOT: PSS.

Intrinsically conductive polymers (ICPs) play a crucial role in enhancing the electrical conductivity of PU foam-based flexible strain sensors. Their conjugated π-electron systems provide inherent electrical conductivity that can be adjusted through doping or structural modifications. In PU foam, ICPs act as conductive fillers by creating percolating networks within the porous structure, enabling electron transport under mechanical deformation. This makes them ideal for applications needing high stretchability. The most common ICPs in this field are polyaniline (PANI), polypyrrole (PPy), and polythiophene (PTh), each with distinct characteristics (Figure 7).

PANI is widely used in PU foam-based strain sensors because of its high electrical conductivity (up to 10^3^ S/cm when doped), simple synthesis, and environmental stability. It can be incorporated into PU foam through in situ polymerization or solution blending, creating conductive pathways that enhance sensor sensitivity [24]. PANI can switch between conductive and non-conductive forms through protonation/deprotonation, allowing precise control of electrical properties. It is also cost-effective and compatible with PU matrices, ensuring even dispersion and strong mechanical properties. However, PANI’s conductivity heavily depends on doping—improper doping can cause reduced performance or instability in varying environmental conditions (e.g., humidity or temperature) [44]. It has poor solubility in common solvents, making processing and integration difficult. Its stiff molecular structure can also decrease the composite’s flexibility, potentially limiting the sensor’s strain range.

PPy is another key ICP for PU foam-based strain sensors, prized for its high conductivity (10–100 S/cm), excellent biocompatibility, and straightforward electrochemical synthesis. Its soft, amorphous nature improves PU foam’s stretchability, making it suitable for large-strain sensing. PPy also bonds well with PU, enhancing sensor durability during repeated use [21]. However, PPy’s main weaknesses are its brittleness and poor long-term stability. Its conductivity decreases over time due to oxidative degradation, especially in humid or oxygen-rich environments. PPy synthesis requires precise control of polymerization conditions, increasing production complexity and cost. Its limited solubility in organic solvents restricts processing options, often requiring in situ polymerization [20].

PTh, especially its derivative poly(3,4-ethylenedioxythiophene) (PEDOT), shows promise for PU foam-based strain sensors due to its high conductivity (up to 10^3^ S/cm for PEDOT) and excellent thermal and chemical stability. PTh derivatives can be modified to improve solubility and processability, allowing uniform incorporation into PU foam. PEDOT forms stable dispersions (e.g., PEDOT: PSS) that enhance the conductivity and flexibility of PU composites [44]. PTh-based sensors show high sensitivity and repeatability, making them ideal for wearable and biomedical applications. However, PTh’s main drawback is its high cost, particularly for derivatives like PEDOT, which limits large-scale production. It needs careful doping to achieve optimal conductivity, as undoped forms conduct poorly. Poor dispersion within PU foam can compromise mechanical properties, causing phase separation or reduced stretchability.

#### 2.2.6. Ionic Liquids (ILs)

**Figure 8:**
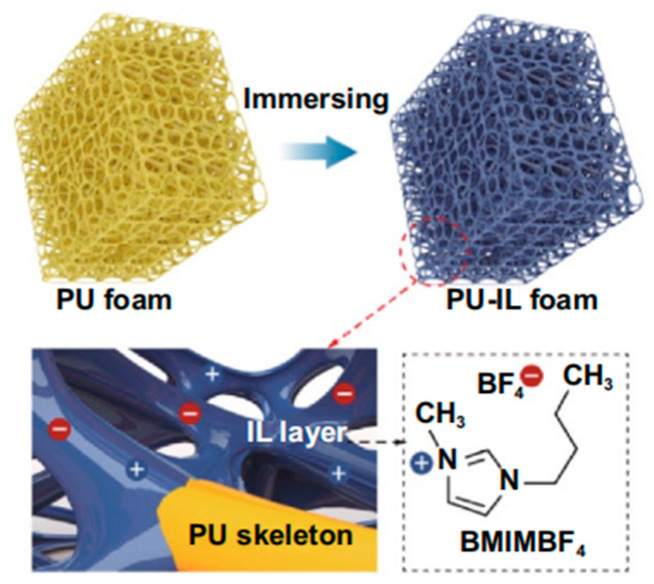
Schematic depicting PU-IL composite foam where ionic liquid coats the PU skeleton surface. Reprinted with permission from ref. [18].

Ionic liquids (ILs) are salts that remain liquid at room temperature, consisting of organic cations paired with organic or inorganic anions. Serving as conductive fillers in flexible strain sensors, they offer high ionic conductivity, low volatility, and electrochemical stability [18] (Figure 8). Their ionic conductivity enables seamless integration with polymer matrices, maintaining sensor functionality during deformation [45]. Unlike traditional metal nanomaterials, such as nanoparticles (AuNPs and AgNPs) and nanowires (AgNWs and AuNWs), or carbonaceous fillers using electron conduction, ILs’ ionic conduction allows better polymer dispersion while preserving mechanical properties. This dual enhancement of flexibility and conductivity makes them ideal for flexible strain sensors. ILs excel in polymer compatibility, preventing dispersion issues and deformation problems, which are common with traditional fillers. Their properties can be customized by modifying cation and anion components, and their stability across various environmental conditions makes them highly versatile.

Carbon nanotubes (CNTs) provide high conductivity and mechanical reinforcement but face dispersion challenges due to agglomeration, addressed via impregnation or electrostatic adsorption methods achieving gauge factors (GF) up to 5.2. Graphene offers exceptional properties yet suffers from sheet stacking and agglomeration, often combined with CNTs to enhance performance. MXenes (e.g., Ti_3_C_2_T_x_) deliver superior conductivity and hydrophilicity for uniform dispersion but incur high synthesis costs. Liquid metals (LMs) enable fluidic adaptability under strain but pose toxicity risks and stability issues. Intrinsically conductive polymers (ICPs)—like PANI (cost-effective but doping-dependent), PPy (flexible but unstable), and PEDOT (high conductivity yet expensive)—balance processability with conductivity limitations. Ionic liquids (ILs) ensure excellent dispersion and flexibility via ionic conduction but differ fundamentally from electron-based fillers. Each filler involves trade-offs: CNTs and MXenes excel in sensitivity, LMs/ILs in stretchability, while graphene hybrids and ICPs offer moderate solutions with distinct cost/performance compromises.

## 3. Fabrication of a Stain Sensor Based on PU Foam

For fabricating strain sensors based on PU foam, introducing conductive fillers into the foam through various methods of embedding or coating is required. The main approaches include dip-coating, in situ polymerization, direct foaming, and the two-step template method.

### 3.1. Dip-Coating Method

Dip-coating involves immersing PU foam in a suspension of conductive materials such as CNTs, MXenes, PEDOT, or LMs. The conductive fillers adhere to the foam surface via physical adsorption or chemical reduction, forming a conductive layer. This method is versatile and easy to implement, but it requires precise control to achieve a uniform and stable coating. A major challenge is ensuring uniform filler distribution, as fillers tend to agglomerate within the polymer matrix [3]. The polar and hydrophobic nature of PU foam, along with the hydrogen bonding sites of urethane groups, makes it compatible with both polar and non-polar fillers. Hydrophilic fillers, however, may require surface modification of the foam to enhance their compatibility. Introducing an intermediate layer, such as polydopamine (PDA), is able to enhance the adhesion, stability, and durability of the coating [21]. The dip-coating process effectively integrates reduced graphene oxide (rGO) and polyaniline (PANI) into the PUF framework, substantially improving its electrical conductivity. Additionally, dip-coating creates micro/nanoscale surface roughness, enabling resistance to common liquids like coffee, milk, and chemical solutions. The incorporation of rGO via dip-coating also enhances flame retardancy. Furthermore, the method improves mechanical stability [46].

### 3.2. In Situ Polymerization

In situ polymerization involves the direct formation of conductive polymers, such as PEDOT, PANI, or PPy, on the PU foam matrix [20,47,48,49]. This method creates a uniform conductive network within the foam, which enhances the sensor’s conductivity and sensitivity. The in situ polymerization technique enhances structural integrity by inhibiting foam expansion during synthesis, improving mechanical robustness [50]. It also provides stronger interfacial bonding compared to coating methods. However, this approach requires careful control of reaction conditions, such as monomer concentration and polymerization time, to ensure uniform coverage and strong adhesion. The complexity of this process may limit its applicability for large-scale production.

### 3.3. Direct Foaming Method

The direct foaming method (one-step in situ foaming) incorporates conductive fillers like CNTs or graphene into the PU prepolymer before foaming. CNTs or graphene are added to the prepolymer mix before foam formation, enabling encapsulation within the cell walls. This integration creates strong interactions between fillers and the PU matrix during foam formation. For instance, graphene-lignin PU foam (Gr-LPUF) composites are created by combining graphene with lignin-based polyols before one-step foaming [24,28]. However, fillers may become embedded within the PU matrix, reducing their surface conductivity. The required surfactants for foam stabilization may also affect conductivity. Optimizing filler concentration and minimizing surfactant use helps balance foam stability and electrical performance.

### 3.4. Two-Step Template Method

The two-step template method involves first creating porous PU foam through template-assisted or phase separation techniques, followed by adding conductive fillers via different ways [5,19,31,33,34,41]. This approach is cost-effective and suitable for large-scale production, resulting in foams with high porosity and good mechanical properties. The main challenge is controlling pore structure uniformity, as variations can lead to uneven filler distribution, which in turn affects sensor performance. Success relies on optimizing both the initial foam structure and the coating process.

Selecting a fabrication method depends on desired sensor properties, conductive filler type, and intended application. Each approach offers unique strengths—dip-coating provides versatility, in situ polymerization ensures uniformity, direct foaming creates strong integration, and the two-step template method enables scalability. While each method faces challenges in adhesion, complexity, or consistency, addressing these through tailored bonding strategies remains essential for creating high-performance PU foam-based strain sensors.

Dip-coating offers versatility and simplicity by immersing PU foam in conductive suspensions (e.g., CNTs, MXenes), but struggles with uniform filler distribution and requires surface modifications (e.g., PDA layers) or foam hydrophilization to enhance adhesion. In situ polymerization grows conductive polymers (e.g., PANI, PEDOT) directly within the foam, ensuring uniform networks and strong interfacial bonding for high sensitivity, though its complex reaction control limits scalability. Direct foaming integrates fillers (e.g., graphene, CNTs) into PU prepolymers before foaming, achieving robust filler-matrix integration but risks embedding fillers too deeply (reducing surface conductivity) and depends on surfactants that may compromise electrical performance. The two-step template method prioritizes cost-effectiveness and scalability by first forming porous PU templates then adding fillers but demands precise pore uniformity to prevent uneven conductive networks. While dip-coating and template methods facilitate large-scale production, in situ polymerization and direct foaming excel in conductivity and integration at the expense of process complexity; ultimately, method selection balances adhesion quality, fabrication simplicity, and application-specific performance needs.

## 4. Sensing Mechanisms

### 4.1. Piezoresistive Sensing Mechanism

Flexible sensors can be grouped into piezoresistive, piezoelectric, and capacitive according to their working principles. Among them, the most commonly used one is the piezoresistive sensor, whose core principle is to convert mechanical deformation (such as pressure and strain) into electrical signal changes [40]. So, the piezoresistive effect expounds the resistance alternation in piezoresistive strain sensor under external pressure, which can be described by Equation (1):

(1) $$\frac{\Delta R}{R_{0}}=(1+2\;\upsilon )\;ɛ+\frac{\Delta \rho }{\rho }$$

The ΔR and *R*_0_ represent the variation in resistance and the initial resistance, respectively; υ stands for Poisson’s ratio, ɛ is strain, while the Δ*ρ* and *ρ* are the resistivity alternation and the original resistivity, respectively. In addition, (1 + 2 υ) *ɛ*, which is the former part, represents the contribution of the external force to the geometric deformation of the sensor, and Δ*ρ/ρ* stands for the contribution of the external force to the resistivity of the material itself [51]. As the core of the sensors, the sensing mechanism of piezoresistive strain sensors can be explained by disconnection mechanisms and the crack effect, etc. Firstly, the disconnection mechanism refers to the fact that conductive materials slip and separate, thus cutting off the conductive path and increasing the resistance when subjected to stress. The other one, the crack effect, refers to the fact that cracks will extend and expand, causing the sensor resistance to increase in the stress concentration area. While after the stress disappears, the cracks will close again and the resistance will return to its initial state [22]. Nevertheless, the sensing mechanisms of sensors usually act together instead of solely. Consequently, the resistance of the strain sensor does not change regularly with the strain. The sensitivity (gauge factor, GF) of the piezoresistive sensor is also an important performance parameter [28],

(2) $$\mathrm{GF}=\frac{\Delta R}{{\varepsilon R}_{0}}$$

*R*_0_ represents the initial resistance; Δ*R* is the change in resistance; *ε* stands for the applied strain, which is usually expressed as the ratio of the length change Δ*L* to the initial length *L*_0_.

Variations in conductive network morphology yield distinct resistance responses under strain, influencing critical sensor indicators including strain range, gauge factor, response dynamics, and reliability. These sensors classify strains as tensile, compressive, bending, or torsional, with their design and conductive structure defining piezoresistive capabilities: wide detection range, high sensitivity, and multi-form strain monitoring. However, these are dependent on external power.

### 4.2. Capacitive Sensing Mechanism

Capacitive sensors are devices that measure pressure by detecting changes in capacitance. According to the parallel capacitive plate Equation (3):

(3) $$C=\frac{\varepsilon A}{d}$$

*ε* is the dielectric constant; *A* represents the effective overlapping area of the plates; *d* is the plate spacing.

Therefore, the applied external force causes changes both in the plate spacing and the effective overlapping area of the plates, resulting in alterations in capacitance. For capacitive pressure sensors, the sensitivity can be described by Equation (4):

(4) $$S=\frac{\partial \frac{∆C}{C_{0}}}{\partial P}$$

*C*_0_ represents the initial capacitance before applying pressure, and *∆C* is the change in capacitance [18].

The sensing mechanism of the capacitor is relatively simple. The sensing mechanism of capacitive sensors mainly lies in their dimensional changes during stretching. Capacitive sensors undergo compression/stretching under external force, exhibiting a distinct linear displacement relationship. Key factors like dielectric layer height and cyclic structure geometry/quantity influence their sensing mechanism. These devices demonstrate rapid force response and high sensitivity, enabling precise low-energy detection of subtle deformations. However, there are limitations including narrow strain range and poor repeatability [41].

### 4.3. Piezoelectric Sensing Mechanism

The piezoelectric pressure sensor is a pressure measurement device which is based on the piezoelectric effect. This phenomenon involves dielectric crystals generating equal but opposite bound charges on their surfaces when mechanical stress (pressure or tension) is applied along specific axes, with charge density proportional to the stress magnitude—termed the “positive piezoelectric effect”. Later, the reciprocal “inverse piezoelectric effect” was confirmed, where applying an electric field induces mechanical deformation in piezoelectric materials, reversible upon field removal. Together, these dual effects enable piezoelectric materials to convert mechanical energy into electrical signals and vice versa [52]. Therefore, the external pressure is transmitted to the piezoelectric crystal through the diaphragm. And the deformation of the crystal causes the separation of charges, generating an instantaneous voltage between the electrodes so that the charges are converted into a measurable voltage or current through the circuit [24].

According to the model proposed by Matteo Beccatelli et al., it can be known that geometrical constraints and Young’s modulus are the key parameters affecting the sensitivity of the sensor and can be described by Equation (5):

(5) $$S=\frac{3{rl}_{0}}{{Et}^{2}}$$

*l*_0_ stands for the thickness of the foam, *r* represents the radius of the internal cells within the material, ^t2^ is the square of the inner wall, and *E* stands for Young’s modulus [48].

Details of some typical PU foam-based strain sensors are summarized in Table 1.

## 5. Applications of PU Foam Strain Sensor

### 5.1. Human Motion Monitoring

Human motion monitoring requires wearable sensors that seamlessly conform to the body and detect movements ranging from subtle pulses from facial expressions to large joint articulations [63]. These sensors must provide high sensitivity, rapid response, and enduring mechanical stability. They need to operate reliably under repeated deformation, detect micro-motions, and maintain comfort during extended use.

The aligned CNTs/TPU foam developed by Huang et al. demonstrates exceptional capabilities in human motion monitoring, leveraging its lightweight, flexible, and highly compressible nature with a density of 0.123 g·cm^3^ and compressibility exceeding 90%. The foam’s aligned porous structure, characterized by ladder-like cells, ensures excellent piezoresistive behavior with linear resistance responses up to 77% strain, enabling precise detection of various human movements. When attached to the sole of a shoe, the foam effectively monitors walking and jumping by registering resistance decreases during compression and recovering to initial values upon release, showcasing high response rates and stability. Similarly, when fixed on the elbow or knee, it accurately detects arm bending and squatting, respectively, with stable and distinct resistance response curves for each motion type. The foam’s ability to maintain reliable performance over 2000 compression-release cycles and its minimal resistance drift (approximately 5% at 60% strain for 1 h) further highlight its durability and suitability [29].

Subsequent advances have focused on enhancing sensitivity-range trade-offs and introducing multifunctionality through hierarchical conductive networks and novel fabrication techniques. Li et al. employed electrospinning of TPU fibers, ultrasonication to anchor CNTs, and rigorous washing to produce a CNT/TPU nanofiber membrane, whose multi-scale network achieved an ultralow detection limit of 0.05%, an ultra-wide strain range of 0.05–600%, 75 ms response time, and durability over 2000 cycles; it uniquely captured both large joint bending and minute signals such as vocal notes and blinking [19]. Yan et al. synthesized a cationic PU via a specialized chain extender and used salt templating with carboxylated CNTs, yielding foams with tensile strength of 12.3 MPa and fracture strains exceeding 1000%; the ionic interactions not only boosted mechanical toughness and sensing stability but also enabled reliable detection of finger, wrist, and even mouth movements in a wearable soft-electronic format [34]. Ren et al. develop a multifunctional piezoresistive pressure sensor inspired by the slit organs of scorpions. The sensor is based on an array narrow orifice configuration (ANOC) and uses hybrid nanofiber decoration with PPy and CNTs on PU foam [60]. This sensor achieves exceptional low-pressure sensitivity (1.8 kPa^−1^ at 1.5 kPa) with rapid response/recovery (120/90 ms) and enduring stability (over 80,000 s), while demonstrating multifunctionality through broad-pressure detection, human posture monitoring, and saline water energy harvesting.

**Figure 9:**
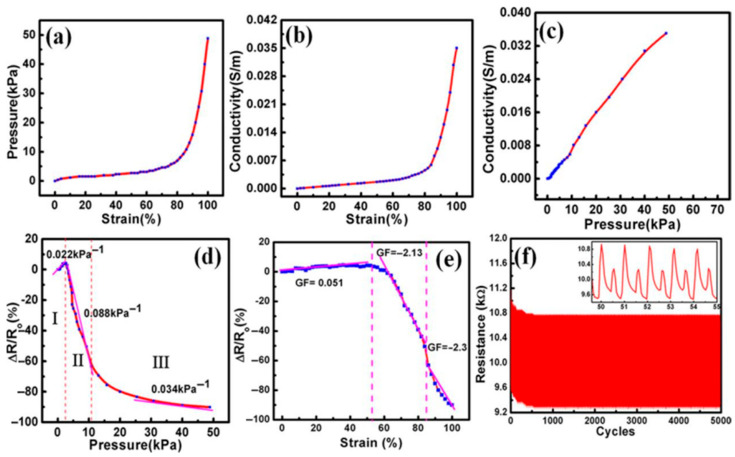
Electrical characteristics of MWCNT/rGO/PU sensor: (**a**) Pressure at different applied strain values, (**b**) conductivity and strain, (**c**) conductivity and pressure, (**d**) change in relative resistance (Δ*R*/*R*_0_) and pressure, (**e**) change in relative resistance and strain, and (**f**) repeatability test of MWCNT/rGO/PU piezoresistive sensor at 50% applied strain for 5000 cycles. Reprinted with permission from ref. [61].

Dip-coating and layer-by-layer assemblies have offered further routes to tune sensitivity zones and compressibility. Tewari et al. developed a low-cost MWCNT/rGO ink for simple dip-coating of commercial PU foam, producing a piezoresistive sensor with three distinct sensitivity regimes (0.022–0.088 kPa^−1^) and 5000-cycle stability (Figure 9), capable of basic word recognition, pulse monitoring, and finger-angle detection typical of early foam sensors [61]. Ma et al. alternated GO and MWCNTs layers on PU via electrostatic layer-by-layer assembly followed by in situ reduction, exploiting a “nanogap disconnection” mechanism at low strain and conductive-skeleton contact at high strain; this approach yielded GF ≈ 1.75 in the 50–75% strain window and supported 75% compressive strain, delivering reproducible finger-bending and arm-contraction sensing [22].

To meet the ever-growing demand for ultra-high sensitivity and broad ranges, hybrid and biomimetic strategies have emerged. Han et al. electrodeposited nickel onto graphene-coated PU sponges and encapsulated them in PDMS, creating Ni/rGO/PU with an extraordinary GF of 3360 over 20–65% strain, sub−100 ms response, and stability beyond 1000 cycles for finger and facial-muscle monitoring [35]. Ren et al. drew inspiration from scorpion cuticles, polymerizing pyrrole within a PDA-modified PU foam to produce a bionic nanofiber network; the resultant PPy/PDA/PU exhibited near-theoretical sensitivity (0.825 kPa^−1^), 120 ms response/recovery, and 1000-cycle durability, enabling precise detection of walking, jumping, yoga postures, and sleep patterns [47].

**Figure 10:**
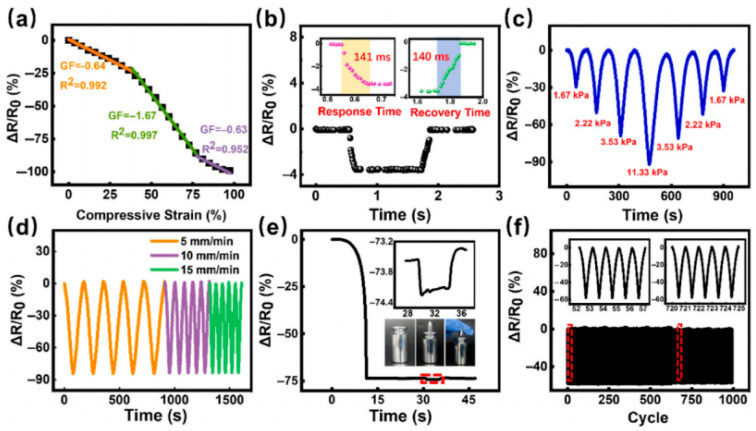
IL/PU Strain sensing performance of ion gel foam sensor. (**a**) The sensor demonstrates a 0–275% resistance response range, with linear curve fitting applied separately across the 0–150% and 150–275% intervals; (**b**) The sensor’s response/recovery times were quantified under 8% tensile strain at 2000 mm min^−1^ stretching velocity; (**c**) Sensor resistance tracking of gradual strain ramping; (**d**) Sensor resistance variation across varying strains; (**e**) Sensor resistance behavior under 35% tensile strain at varying stretching rates; (**f**) The sensor undergoes over 1000 long-term durability tests with a maximum strain of 40%. The illustrations, respectively, represent the resistance responses of the sensor within the cycles of 50–54 and 785–789. Reprinted with permission from ref. [45].

The integration of multifunctional arrays and ionic gels has pushed PU foams toward smart e-skin platforms. Adepu et al. fabricated a 5 × 5 sensor matrix by dip-coating MXene on PU foam for pressure/strain and on cellulose paper for temperature, achieving pressure sensitivity of 34.24 kPa^−1^, GF ≈ 323.6 (5–20% strain), and real-time gesture recognition (“BITSH”), with applications spanning motion capture to security and interactive education [6]. Luo et al. combined chemical foaming with ultrasound-assisted ionic-liquid loading to create an ionic-gel PU foam displaying 99% compressibility, 275% stretchability, dual pressure/strain sensing down to 0.17 kPa, 200 ms response, and 8 × 8 pressure-imaging capability (Figure 10)—realizing multifunctional sensing within a single-material architecture [45].

While PU foam sensors demonstrate impressive capabilities in detecting motions ranging from subtle physiological signals (e.g., pulses, blinking) to large joint articulations—achieving high sensitivity (e.g., GF up to 3360), ultralow detection limits (0.05%), and wide strain ranges (up to 600%)—their real-world utility faces significant limitations. Practical adoption is hindered by inconsistent durability claims (e.g., cycle stability tests often exclude environmental variables like humidity/temperature), calibration drift under prolonged use (e.g., ~5% resistance drift in 1 h), and oversimplified demonstrations (e.g., controlled lab settings for finger bending vs. complex real-world movements). Multifunctionality claims (e.g., energy generation or multimodal sensing) frequently prioritize novelty over reliability, lacking rigorous validation for concurrent operation. Moreover, scalability remains questionable: complex fabrication (e.g., electrospinning, biomimetic designs) increases cost, while sensor arrays struggle with signal crosstalk and integration into ergonomic wearables. Although innovations, like aligned CNT networks or ionic gels, enhance compressibility/stretchability, unresolved challenges in power efficiency, wireless data transmission, and long-term stability under physiological conditions (e.g., sweat, skin adhesion) underscore a persistent gap between laboratory prototypes and deployable health-monitoring systems.

### 5.2. Medical and Healthcare Applications

In the realm of medical and healthcare applications, flexible strain sensors are increasingly vital for continuous physiological monitoring, early disease detection, and personalized therapeutic interventions. These sensors must exhibit high sensitivity, rapid response times, biocompatibility, and mechanical robustness to effectively interface with the human body and accurately capture subtle physiological signals.

**Figure 11:**
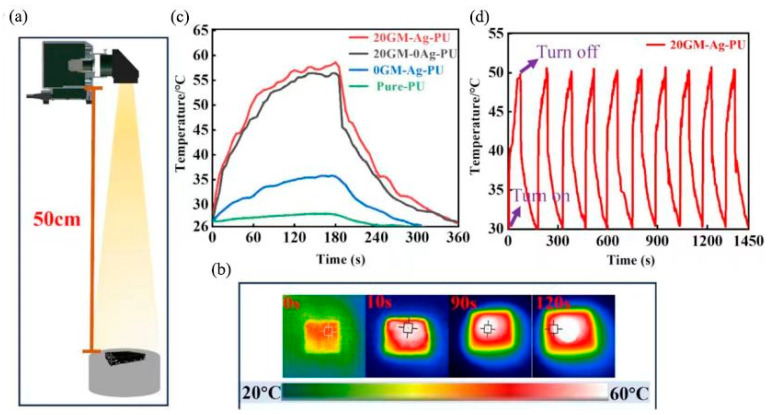
Foam photothermal energy conversion performance: (**a**) Diagram illustrating light-to-heat conversion in GM-Ag-PU foam under simulated sunlight; (**b**) IR images tracking 20GM-Ag-PU foam under simulated sunlight over 120 s; (**c**) Temperature alternation in Pure-PU, 0GM-Ag-PU, 20GM-0Ag-PU, 20GM-Ag-PU foams monitored throughout the heating and cooling processes; (**d**) Repeated heating-cooling assessment for 20GM-Ag-PU cellular polymer. Reprinted with permission from ref. [53].

One notable advancement involves the integration of silver nanoparticles (Ag NPs) and vertically aligned graphene into PU foam through ultrasonic irradiation [53]. This composite material exhibits exceptional antibacterial properties, achieving 100% inhibition of *Escherichia coli*, and demonstrates efficient photothermal conversion, reaching temperatures up to 58.6 °C (Figure 11). Such multifunctionality—combining sensing, antibacterial, and photothermal capabilities—positions this material as a promising candidate for medical wearable devices aimed at infection control and therapeutic applications.

In addressing respiratory monitoring, Brady et al. developed a smart fabric by in situ polymerizing PPy on PU foam. This conductive composite material responds to different breathing patterns by exhibiting changes in electrical resistance, indicating its potential for tracking respiratory amplitude. However, as an early-stage development, the sensor faces challenges such as low sensitivity (~0.0007 mS/N), slow response time (~50 s), significant hysteresis, limited stability, and baseline conductivity drift, necessitating further optimization for clinical applications [20].

Advancements in gait analysis and neurological disorder detection have been achieved by Chen et al., who fabricated an anisotropic MXenes/PDA/TPU composite foam using directional freezing and dip-coating techniques [21]. The resulting sensor exhibits enhanced sensitivity (GF of 2.36), a rapid response time of 40 milliseconds, and increased compressive strength by 48.1%. When integrated into smart insoles, this sensor effectively monitors walking postures, offering the potential for early diagnosis of conditions like Parkinson’s disease.

To overcome limitations in sensitivity at high voltages, Zheng et al. constructed a three-dimensional interconnected porous TPU framework embedded with liquid metal electrode layers and MXene films serving as high dielectric constant layers [41]. This capacitive sensor performs a sensitivity of 1.91 kPa^−1^ within a detection range up to 260 kPa, maintains fast response times (60 ms loading and 110 ms unloading), and demonstrates stability over 4000 cycles. Such performance is critical for applications like radial artery blood pressure wave detection and human–computer interaction interfaces.

**Figure 12:**
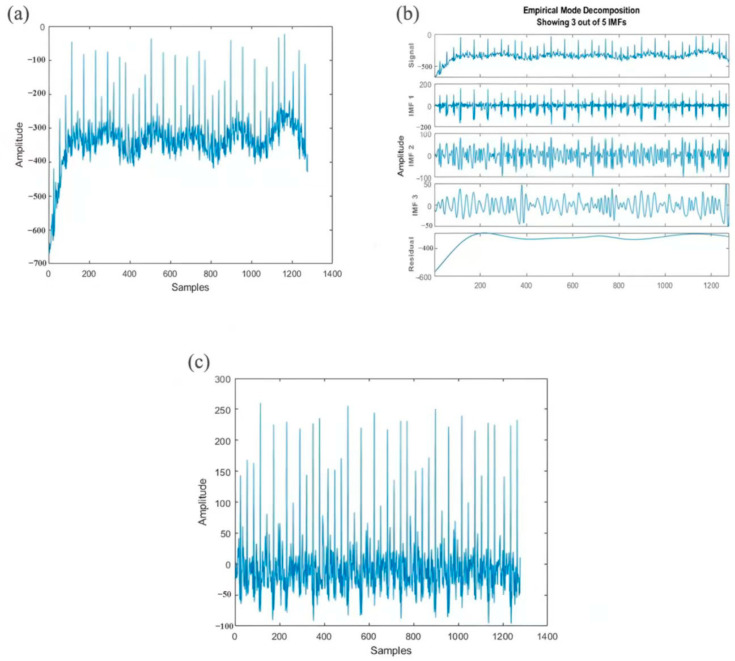
Electrocardiogram signals were collected using Ag/AgCl electrode patches. (**a**) Real-time electrocardiogram signal. (**b**) Electromagnetic radiation level. (**c**) Reconstruct the signal. Reprinted with permission from ref. [24].

In the domain of cardiac monitoring, Rajeev et al. developed a flexible dry electrode by integrating PANI, MWCNTs, and ZnO into castor oil-based PU foam through in situ polymerization [24]. This electrode exhibits high conductivity (1817 S/m) and excellent biocompatibility, enabling accurate electrocardiogram (ECG) signal acquisition with R-wave accuracy exceeding 99%. The synergy between conductive polymers and nanofillers ensures both flexibility and high conductivity, making it a viable alternative to traditional gel electrodes (Figure 12).

Addressing the need for high sensitivity in low-pressure ranges, Yao et al. introduced a fractured microstructure design in rGO-coated PU foams [39]. This design enhances piezoresistive sensitivity by two orders of magnitude within the 0–2 kPa pressure range, enabling real-time heart rate monitoring and spatial pressure mapping through a 13 × 11 pixel array. The sensor maintains accuracy even under bending conditions, highlighting its potential in artificial skin, biological signal monitoring, and sound sensing applications.

**Figure 13:**
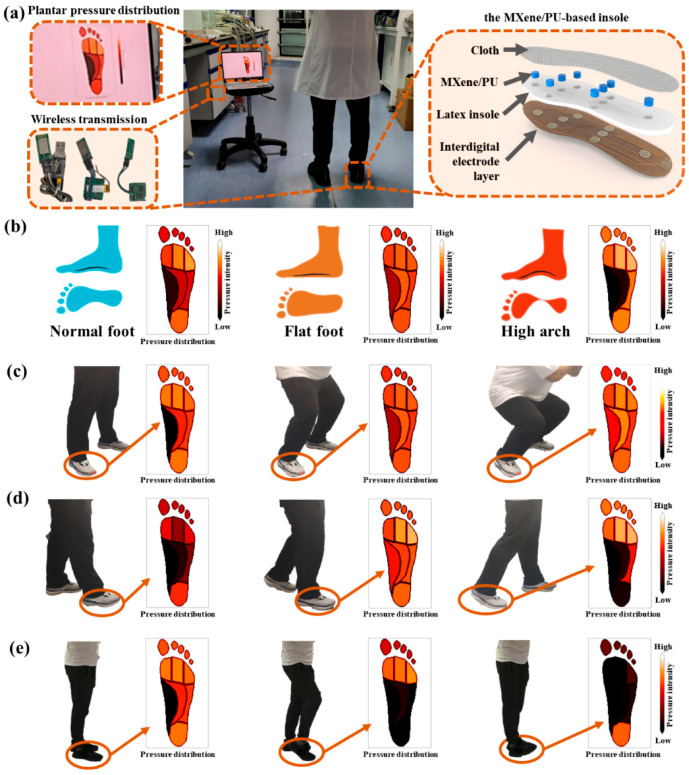
Foot health application: MXene/PU pressure sensor. (**a**) The practical application scenarios, composition, and schematic diagram representation of the wireless wearable foot monitoring system. (**b**) The schematic diagram for the identification and assessment of normal feet, flat feet, and high arches. (**c**) Real-time foot force distribution analysis for subjects exhibiting flexible flat feet during full squatting and (**d**) walking, and (**e**) the dynamic plantar pressure mapping in subjects with normal foot when different parts of the feet touch the ground. Reprinted with permission from ref. [27].

In foot health monitoring, Wang et al. utilized an iron foam template method to construct a 3D microporous MXene/PU foam [27]. This composite combines the high electrical conductivity of MXene with the mechanical stability of PU, facilitating real-time pressure distribution monitoring on the sole. The sensor effectively distinguishes between normal feet, flat feet, high arches, and dynamic flexible flat feet, offering significant potential in medical rehabilitation, gait analysis, and human–computer interaction (Figure 13).

Wang et al. developed a lignin-based PU foam by liquefying lignin into polyol and incorporating MWCNTs through a foaming method [28]. The sensor exhibits excellent sensitivity and environmental stability, detecting both large-scale limb movements and subtle physiological signals such as swallowing, breathing, pulse, and throat vibrations. The sensor can also detect non-contact movements like blowing or vibrations, making it suitable for diverse healthcare monitoring scenarios. Due to its biodegradability and the ability to recycle the conductive MWCNTs, the sensor is environmentally friendly, offering great promise for future use in flexible wearable health devices.

While PU foam sensors show promising capabilities in medical monitoring—demonstrating high sensitivity for physiological signals (e.g., ECG R-wave accuracy > 99%), antibacterial functionality, and specialized designs for gait analysis or pressure mapping—their clinical translation faces unresolved challenges. Many prototypes prioritize technical novelty over practical viability, overlooking critical factors like long-term biocompatibility validation (e.g., cytotoxicity of Ag NPs/MXenes), sterilization compatibility, or drift resistance in humid/moving environments (e.g., respiratory sensors exhibit hysteresis and baseline drift). Scalability is hampered by complex fabrication (e.g., directional freezing, liquid metal infusion) that increases costs, while multifunctional claims (e.g., photothermal therapy with sensing) lack integrated validation in physiologically relevant conditions. Environmental sustainability assertions (e.g., “biodegradable” lignin-PU foams) rarely address end-of-life nanofiller (MWCNTs) recovery or toxicity. Crucially, clinical applicability is undermined by insufficient human trials, regulatory pathway considerations, and poor interoperability with existing medical systems—evident in smart insole prototypes that detect foot pathologies but lack real-time diagnostic algorithms or EHR integration. Despite impressive sensitivity metrics, these limitations highlight a significant gap between lab-scale achievements and deployable, patient-centric healthcare solutions.

### 5.3. Industrial and Environmental Sensing

Deformation and pressure monitoring in industrial and environmental applications necessitate sensors that are not only sensitive and responsive but also robust and adaptable to challenging environments. PU foam-based flexible strain sensors have emerged as promising candidates to meet these demands due to their inherent flexibility, lightweight nature, and tunable mechanical properties.

A notable development in this field is the multifunctional integrated sensor designed by Hong et al., which combines MWCNTs and PANI-coated PU foam. This sensor is capable of simultaneously detecting pressure, temperature, and ammonia gas, achieved through the synergistic effects of piezoresistive, thermoelectric, and gas adsorption mechanisms. It achieves a 2.1 kPa^−1^ pressure sensitivity with 20 ms response speed and enduring stability through 10,000 cycles, making it highly suitable for continuous monitoring in industrial settings [49]. Nanostructured polymer foams have also gained traction for their tailored properties. Eghbalinia et al. developed a semi-interpenetrating network (semi-IPN) by impregnating PU foams with room temperature vulcanized silicone rubber (RTVSR) and MWCNTs [23]. Adjusting the crosslinking density optimized the sensor’s compressibility and sensitivity, achieving a GF of 3.71 within 60% compressive strain. This moderate sensitivity and stability position the material as a viable candidate for pressure sensors in applications where consistent performance under moderate strain is needed.

In another approach, Shi et al. enhanced the mechanical and electrical properties of PU foam by coating it with rGO through a self-assembly method. By optimizing the pore size between 0.5 and 1.0 mm, they achieved a balance between elasticity and conductivity. Under large strain conditions, the sensor’s resistance changes by three orders of magnitude (90% strain), demonstrating its potential for large deformation monitoring in industrial applications [38]. Fei et al. extended these concepts by incorporating MWCNTs via solution blending followed by ScCO_2_ microcellular foaming, achieving stable performance over 1000 compression cycles, a low energy-loss coefficient, and a gauge factor of 1.88 at 35% strain; their MWCNTs/TPU foam delivered consistent current responses during real-time pedaling tests but remained limited to sub−50% strain sensing [33]. Liu et al. created porous CNT/TPU conductive nanocomposites using solution-blending and TIPS methods to develop highly effective piezoresistive sensors. These sensors showcase remarkable compressibility, withstanding up to 90% strain, while maintaining good electrical conductivity with just 0.51 vol.% CNT loading. They feature rapid response times and excellent recovery properties, performing consistently across a wide strain range [31]. They also developed a graphene/TPU foam using TIPS [30]. With a porosity of 90%, this foam reduces density while significantly boosting compressibility, achieving a 110% increase in compressive strength and supporting up to 90% strain. The incorporation of graphene stabilizes electrical conductivity at 10^−1^ S/cm with a low percolation threshold of 0.061 vol%. During compression, the breakdown of cell walls leads to unique positive piezoresistive behavior, with a sensitivity of approximately 12.24 at strains exceeding 60%.

Beyond the strain range, rapid response to mechanical changes is essential for many sensing applications. Li et al. advanced this aspect by impregnating PU foam with sodium alginate (SA) and rGO to create a highly responsive sensor [36]. The optimized pore structure, enhanced by SA, delivers a response time of 100 ms and a recovery rate of 98%, enabling real-time pressure switching. Such properties are critical for applications requiring immediate feedback, such as tactile sensors in robotics or safety systems that demand fast reaction times.

**Figure 14:**
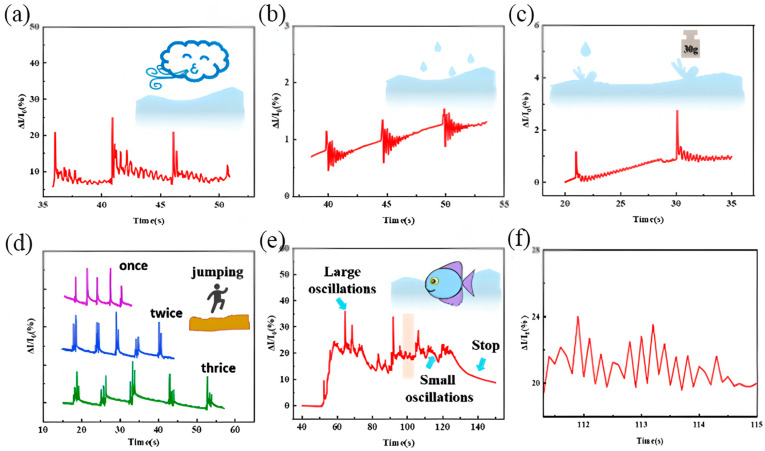
Underwater sensing performance of (**a**) simulating wind, (**b**) water droplet fall, (**c**) falling of different weights, (**d**) detecting jumping human bodies on the ground, (**e**) simulating fish swimming, and (**f**) local amplification of electrical signals simulating fish swimming. Reprinted with permission from ref. [59].

For applications in underwater environments, Zhao et al. developed a lignin-based PU foam composite with superhydrophobic properties. This material, achieved through a one-step foaming process, exhibits a water contact angle of 154.2°, making it ideal for underwater sensors that require resistance to water and oil. Its application extends to micro-vibration detection and rescue communication, showcasing its versatility in complex industrial environments [59] (Figure 14).

Peng et al. developed LM/PU foams using a novel in situ foaming technique that combines liquid metals with PU to create materials with exceptional properties [42]. Under pressure, the LM forms dynamic conductive pathways, yielding a high-pressure sensitivity (GF > 25) and a rapid response time of 202 ms. The foam fully rebounds at 95% compressive strain and has been validated in impact tests for vehicle alarm systems. This work marks a pioneering achievement in combining high electrical conductivity with superelasticity, enhancing the material’s potential for real-time resistance detectors and flexible electronic components. Cai et al. explored a different approach by embedding liquid metal microdroplets into PU foam [43]. By exploiting the solid–liquid phase transition of gallium (melting point ≈ 29.76 °C), they created flexible composite sponges with a 90% elastic recovery rate and a pressure sensing range up to 386.8 kPa, which shows a broad pressure range. Addressing the challenges of liquid metal leakage and conductive network stability, Huang et al. modified PU sponges with PDA and liquid metal nanoparticles. This modification formed a continuous conductive layer with an electrical conductivity of 478 S·cm^−1^ and a minimal resistance change rate of 2% at 50% strain [57]. Further enhancing the functionality of PU foam-based sensors, Zhang et al. introduced microcellular thermoplastic polyurethane LMs/TPU composite foams [32]. By employing a water-vapor-induced phase separation process, they achieved a microporous structure that suppresses liquid metal leakage and improves dispersion uniformity. These composites not only offer high stability for deformation monitoring but also provide electromagnetic shielding capabilities, with an efficiency of 49.5 dB, indicating their potential in applications requiring electromagnetic interference protection.

**Figure 15:**
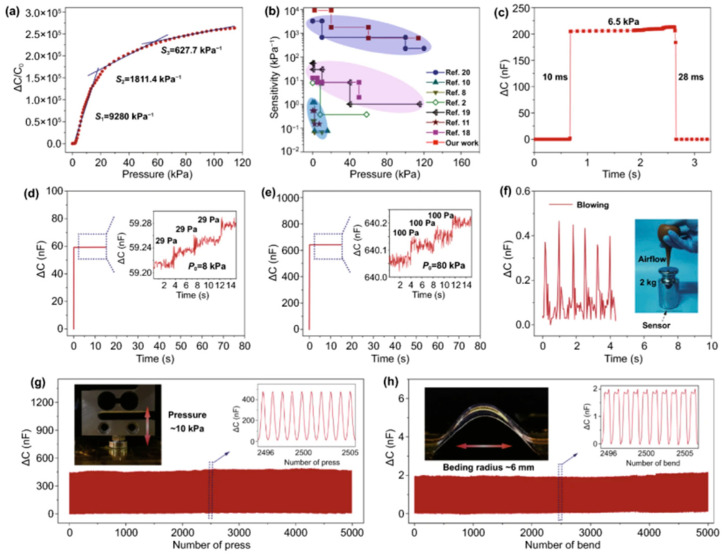
The sensing performance of ILs/PU foam capacitive pressure sensors. (**a**) This sensor exhibits a maximum sensitivity of 114 kPa. (**b**) Comparative analysis of device sensitivity versus existing literature data. (**c**) Pressure sensor temporal response characteristics. Detect the weak pressure when the preload pressure is 8 kPa (**d**) and 80 kPa (**e**). (**f**) Capacitance variation in this sensor monitored under 127 kPa base pressure with 2 kg weight during airflow exposure. (**g**) Sustained pressure-discharge performance through 5000 cycles (10 kPa peak), (**h**) alongside H-bending release stability exceeding 5000 cycles at ~6 mm curvature. Reprinted with permission from ref. [18].

**Figure 16:**
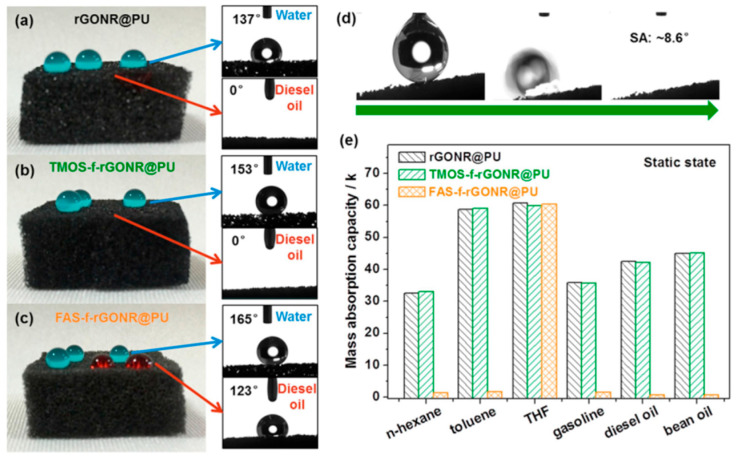
Surface hydrophobicity of three composites: (**a**) rGONR@PU, (**b**) TMOS-f-rGONR@PU, (**c**) FAS-f-rGONR@PU, (**d**) FAS-f-rGONR@PU water sliding phenomena, (**e**) comparison of mass oil absorption capacity of different samples at static state. Reprinted with permission from ref. [56].

Liu et al. focused on capacitive pressure sensors by impregnating high-porosity PU foam with ILs [18]. This simple impregnation process resulted in a continuous three-dimensional conductive network, achieving a maximum sensitivity of 9280 kPa^−1^ (Figure 15). The sensor’s low Young’s modulus of 3.4 kPa and high porosity (95.4%) contribute to its excellent compressibility and contact area response, making it suitable for applications such as underwater motor vibration and mechanical fault monitoring. Qiang et al. utilized a dip-coating method to combine silane-functionalized graphene oxide nanoribbons with PU foams [56]. This composite material exhibits a surface contact angle of 165° (Figure 16), low resistance of 82.8 kΩ, and excellent mechanical elasticity, maintaining stable performance after 100 cycles under 80% compressive strain. The sensor’s selectivity for oil and solvent targets makes it particularly suitable for marine crude oil spill control, highlighting its application in environmental monitoring and protection.

The incorporation of graphene has further enriched the capabilities of PU foam sensors. Ugarte et al. prepared graphene sheets through liquid exfoliation and dispersed them into PU foam via ultrasound-assisted impregnation [54]. They observed a three-stage resistance response with increasing strain: a sharp drop at low strain due to enhanced graphene contact, stability at medium strain as the pore structure collapses, and another drop at high strain as the material densifies. This unique behavior suggests applications in flexible sensors and pressure-sensitive components, where varying strain levels can be leveraged for diverse sensing tasks. Future efforts could refine graphene dispersion and enhance the mechanical-electrical synergy.

Simplifying fabrication while ensuring stability is crucial for scaling up sensor production. Zhong et al. tackled this by producing conductive rGO/PU foam composites through a straightforward soaking process followed by hydrazine hydrate vapor reduction [55]. This method yields sensors with high sensitivity (0.17 kPa^−1^), a wide pressure detection range (0–25 kPa), a fast response (300 ms), and excellent cycle stability. By overcoming the complexity and stability drawbacks of traditional piezoresistive sensors, this approach offers an efficient, cost-effective solution for flexible piezoresistive sensors. Future research could investigate its durability in extreme conditions and integration with other electronic systems.

While PU foam sensors demonstrate compelling versatility for industrial and environmental monitoring—achieving multifunctionality (e.g., simultaneous pressure/temperature/gas detection), extreme compressibility (up to 95% strain), and environmental resilience (e.g., superhydrophobicity for underwater use)—their real-world deployment faces unresolved scalability and reliability challenges. Many high-performance claims (e.g., GF > 25 for LM/PU composites or 9280 kPa^−1^ sensitivity for IL-impregnated foams) derive from idealized lab conditions, overlooking critical field variables like temperature fluctuations, chemical exposure, or cyclic fatigue in dynamic industrial settings. Fabrication complexity (e.g., in situ foaming, ScCO_2_ processing, or liquid metal encapsulation) escalates costs and hinders mass production, while nanomaterial integration (e.g., MXenes, rGO) introduces unresolved environmental risks, such as nanofiller leakage during oil-spill monitoring or end-of-life disposal of non-biodegradable composites. Although innovations like lignin-based foams or LM leakage suppression offer sustainability promises, they lack lifecycle assessments verifying durability under prolonged stress or harsh environments. Crucially, sensor networks for infrastructure or environmental monitoring remain hampered by unaddressed power constraints, wireless integration barriers, and calibration drift—evident in marine oil sensors that detect solvents but omit biofouling resistance or long-term salinity impact studies. These gaps underscore a disconnect between academic performance metrics and field-ready robustness needed for industrial adoption.

## 6. Summary and Outlook

PU foam-based flexible strain sensors have emerged as a transformative technology, capitalizing on their low modulus, high porosity, and tunable mechanical properties to meet the demands of next-generation wearable electronics and biomedical applications. Recent advancements have significantly enhanced their capabilities, with the integration of multifunctional sensing—detecting pressure, temperature, and chemical stimuli simultaneously—broadening their practical utility. The incorporation of advanced conductive nanomaterials, such as CNTs, MXenes, and graphene derivatives, into PU foam matrices has markedly improved sensitivity and response time, enabling applications like motion and biomedical monitoring. Moreover, a growing emphasis on sustainable development, exemplified by the use of biomass-based materials and recyclable components, aligns these sensors with eco-friendly innovation trends. Biomass-based PU systems such as lignin-based foams offer renewable pathways but face challenges in lifecycle management. Filler leaching, especially from ILs or metallic particles, can pose environmental hazards. Further, while MWCNTs or MXenes improve performance, their synthesis is energy-intensive. A life-cycle CO_2_ footprint analysis of conductive filler production and recycling is thus essential for large-scale deployment.

Despite these strides, critical challenges persist. Long-term stability and durability remain elusive under fluctuating environmental conditions, such as humidity, temperature variations, and mechanical stress, which can degrade sensor reliability over time. Humidity exposure causes protonation changes in PANI, reducing its conductivity over time due to water-induced dedoping. This affects long-term stability, especially in wearable sensors operating in perspiration-rich environments. Additionally, while laboratory-scale prototypes exhibit impressive performance, scaling up production to achieve consistent quality and cost-effectiveness poses a significant hurdle, limiting commercial viability. These issues underscore the need for continued innovation in materials design and fabrication techniques.

Looking forward, the future of PU foam-based sensors lies in overcoming these barriers while pushing the boundaries of functionality and sustainability. Optimizing advanced fabrication methods, such as 3D printing and self-assembly, promises to enhance reproducibility and scalability. Exploring novel conductive fillers or hybrid nanocomposites could further elevate sensor performance. The development of multifunctional sensors capable of detecting combined stimuli—pressure, temperature, and biochemical signals—holds particular promise for personalized healthcare, enabling real-time monitoring of chronic conditions. The integration of artificial intelligence and machine learning into the manufacturing and operational frameworks of PU foam-based sensors represents a transformative frontier for enhancing functional intelligence. Machine learning algorithms unlock advanced functionalities in deployed sensors. For instance, they can process complex, multi-modal sensor data (e.g., simultaneous pressure, strain, and temperature signals) to extract meaningful patterns, suppress noise, and detect anomalies. In healthcare applications, machine learning models trained on physiological datasets enable real-time interpretation of subtle biomechanical signals (e.g., distinguishing pathological gait from normal motion or predicting cardiovascular events from pulse waveforms). Similarly, for industrial sensing, artificial intelligence facilitates predictive maintenance by correlating strain data with equipment degradation models. Furthermore, PU composites with self-healing agents or reversible dynamic bonds are being developed for durability extension and lifecycle improvement. Additionally, biocompatible and biodegradable sensors could revolutionize medical applications by improving bio-integration.

In conclusion, PU foam-based flexible strain sensors are poised for transformative impacts in a wide range of fields, from healthcare and wearable electronics to industrial monitoring and robotics. By addressing the current challenges and advancing interdisciplinary collaboration, these sensors will continue to evolve, offering new opportunities for smart, responsive, and adaptable technologies in the future.

## Figures and Tables

**Table 1:** Main features of various flexible PU foam-based strain sensors.

Conductive Material	Materials	Fabrication Method	Sensitivity/GF (at Strain %)	Sensing Range	Response/Recovery Time (ms)	Durability (Cycles at Strain %)	Ref.
CNTs	MWCNTs/PU	dip-coating	3.51 (70%)	0–90%	/	20 (50%)	[23]
	MWCNTs-C/TPU	template-assisted method and dip-coating	5.38 (0–30%)	0–782%	200	40 (30%)	[34]
	MWCNTs/TPU	template-assisted method	1.22 (0–77%)	0–77%	/	2000 (30%)	[29]
	MWCNTs/TPU	solution-blending and ScCO_2_ foaming	1.88 (35%)	0–50%	/	1000 (30%)	[33]
	CNTs/TPU	electrospinning and dip-coating	0.101 (400%)	0.05–600%	75	2000 (50%)	[19]
	MWCNTs/Ag/TPU	salt-template-assisted method and dip-coating	1.4 (0–55%)	0–70%	20/20	500 (50%)	[5]
	CNTs/TPU	solution-blending and TIPS	/	0–90%	/	50 (90%)	[31]
MXenes	MXenes/PU	dip-coating	323.59 (5–20%)	0–20%	510/65	2500 (10%)	[6]
	MXene/PU	iron foam template and dip-coating	0.96144 kPa^−1^	0–454.7 kPa	140/152	15,000 (130 kPa)	[27]
	MXenes/PDA/TPU	directional freezing and dip-coating	2.36 (2.5–20%)	0–80%	40	5000 (50%)	[21]
	MXenes/CS/PU	dip-coating	3 (45–85%)	0–85%	19	5000 (20%)	[40]
Gr	Gr/TPU	TIPS	12.24 (60–90%)	0–99%	/	50	[30]
	rGO/PU	dip-coating	/	0–100%	100/740	/	[38]
	rGO/BaTiO_3_/PU	dip-coating	2.64 kPa^−1^	0–60 kPa	560	/	[37]
	rGO/SA/PU	direct foaming method	/	0–40%	100/100	100 (50%)	[36]
	GO/Ag/PU	dip-coating and chemical reduction of Ag	66.3 (45–60%)	0–60 kPa	/	100 (15%)	[53]
	Gr/PU	dip-coating	/	0–70%	/	/	[54]
	rGO/PU	dip-coating	0.26 kPa^−1^	0–10 kPa	/	10,000 (2 kPa)	[39]
	rGO/PU	dip-coating	0.17 kPa^−1^	0–25 kPa	300/300	50(3.125 kPa)	[55]
	rGO/PU	dip-coating	/	0–80%	/	100 (80%)	[56]
	Ni/GO/PU	dip-coating and electrodeposition of Ni	3360.09 (20–65%)	0–65%	100	1000 (30%)	[35]
IL	IL/PU	dip-coating	9280 kPa^−1^	0–120 kPa	10/28	5000(10 kPa)	[18]
	IL/PU	dip-coating	2.82 (150–275%)	0–275%	141/140	1000 (40%)	[45]
ICP	PPy/PU	in situ polymerization	/	0–100%	50,000	/	[20]
	PPy/PDA/PU	in situ polymerization	6.66 (0–20%)	0–70%	120/100	1000	[47]
	PEDOT-PSS/PU	dip-coating	0.3 kPa^−1^ (0–30 kPa)	0–50 kPa	/	/	[48]
	PTh-Ag/PU	in situ polymerization	152.24 (60–80%)	0–80%	/	1000 (30%)	[44]
LMs	Ga/PU	dip-coating	/	64.5–386.8 kPa	/	1000 (174.5 kPa)	[43]
	LMs/PU	direct foaming method	35.8 (19–22%)	0–100%	202	800 (10%)	[42]
	LMs/PDA/PU	dip-coating	/	0–150%	/	1000	[57]
	LMs/TPU	WVIPS	/	0–741%	/	250 (200%)	[32]
Multiple conductive fillers	GO/PPy/PU	dip-coating and in situ polymerization	2.1 (0–40)	0–80%	70	10,000 (45%)	[58]
	PPy/GO/PU	dip-coating and in situ polymerization	13.89 (70–90%)	0–90%	100	2000 (50%)	[59]
	PPy/CNT/PU	dip-coating and in situ polymerization	1.8 kPa^−1^ (0–1.5 kPa)	10–70%	120/90	1000 (40%)	[60]
	LM/MXene/TPU	template-assisted method and dip-coating	1.91 kPa^−1^	0–260 kPa	60/110	4000 (50%)	[41]
	MWCNT/PANI/PU	dip-coating	2.1 kPa^−1^	0.05–30 kPa	20	10,000 (1 kPa)	[49]
	MWCNTs/rGO/PU	dip-coating	2.3 (100%)	0–100%	30	5000 (50%)	[61]
	MWCNTs/rGO/PU	dip-coating	1.75 (50–75%)	0–75%	/	/	[22]
	MXenes/MWCNTs/TPU	salt-template-assisted method and dip-coating	363 (80–101%)	0–101%	/	200 (80%)	[62]

## Data Availability

No new data were created or analyzed in this study. Data sharing is not applicable to this article.

## Abbreviations

The following abbreviations are used in this manuscript:

3D	Three-dimensional
Ag NPs	Silver nanoparticles
Ag NWs	Silver nanowires
ANOC	Array narrow orifice configuration
Au NPs	Gold nanoparticles
Au NWs	Gold nanowires
CNTs	Carbon Nanotubes
CS	Chitosan
ECG	Electrocardiogram
FAS-f-rGONR	(heptadecafluoro-1, 1, 2, 2-tetradecyl) trimethoxysilane functionalized rGONR
GF	Gauge Factor
GM	Multilayered graphene
GO	Graphene Oxide
Gr	Graphene
Gr-LPUF	Graphene-Lignin PU foam
ICPs	Intrinsically conductive polymers
IL	Ionic Liquid
IR	Infrared Spectroscopy
LM	Liquid Metal
MWCNTs	Multiwalled Carbon Nanotubes
MWCNTs-C	Carboxylic Multiwalled Carbon Nanotubes
PANI	Polyaniline
PDA	Polydopamine
PDMS	Polydimethylsiloxane
PEDOT	Poly(3,4-ethylene dioxythiophene)
PPI	Pores per inch
PPy	Polypyrrole
PTh	Polythiophene
PU	Polyurethane
rGO	Reduced Graphene Oxide
rGONR	Reduced Graphene Oxide Nanoribbons
RTVSR	Room temperature vulcanized silicone rubber
SA	Sodium alginate
ScCO_2_	Supercritical Carbon Dioxide
semi-IPN	Semi-interpenetrating network
TIPS	Thermally Induced Phase Separation
TMOS-f-rGONR	Trimethoxyoctadecylsilane functionalized rGONR
TPU	Thermoplastic Polyurethane
WVIPS	Water-Vapor-Induced Phase Separation
